# HIV interactions with monocytes and dendritic cells: viral latency and reservoirs

**DOI:** 10.1186/1742-4690-6-51

**Published:** 2009-06-01

**Authors:** Christopher M Coleman, Li Wu

**Affiliations:** 1 Department of Microbiology and Molecular Genetics, Medical College of Wisconsin, 8701 Watertown Plank Road, Milwaukee, WI 53226, USA

## Abstract

HIV is a devastating human pathogen that causes serious immunological diseases in humans around the world. The virus is able to remain latent in an infected host for many years, allowing for the long-term survival of the virus and inevitably prolonging the infection process. The location and mechanisms of HIV latency are under investigation and remain important topics in the study of viral pathogenesis. Given that HIV is a blood-borne pathogen, a number of cell types have been proposed to be the sites of latency, including resting memory CD4^+ ^T cells, peripheral blood monocytes, dendritic cells and macrophages in the lymph nodes, and haematopoietic stem cells in the bone marrow. This review updates the latest advances in the study of HIV interactions with monocytes and dendritic cells, and highlights the potential role of these cells as viral reservoirs and the effects of the HIV-host-cell interactions on viral pathogenesis.

## Background

Human immunodeficiency virus (HIV) remains a devastating human pathogen responsible for a world-wide pandemic of acquired immunodeficiency syndrome (AIDS). Despite extensive research of HIV since the virus was identified over 25 years ago, eradication of HIV-1 infection and treatment of AIDS remain a long-term challenge [1,2]. The AIDS pandemic has stabilised on a global scale. In 2007, it was estimated that 30 to 36 million people world-wide were living with HIV, and 2.7 million people were newly infected with HIV. Moreover, AIDS-related deaths were increased from an estimated 1.7 million people in 2001 to 2.0 million in 2007. Africa continues to be over-represented in the statistics, with 68% of all HIV-positive people living in sub-Saharan countries. The young generation represents a large proportion of newly infected population who may contribute to the overall spread of HIV in the future [3].

There are two types of HIV, HIV-1 and HIV-2; both are capable of causing AIDS, but HIV-2 is slightly attenuated with regards to disease progression [4]. Given the relative severity of HIV-1 infection, the majority of studies have been done using HIV-1. The infection dynamics of HIV-1 are very interesting. Upon initial HIV-1 infection, there is a period of continuous viral replication and strong immune pressure against the virus, resulting in a relatively low steady state of viral load. The virus then enters a chronic stage, wherein there is limited virus replication and no outward signs of disease. This clinical phase can last many years, ultimately leading to destruction of the host immune system due to chronic activation or viral replication. This results in the onset of the AIDS stage with opportunistic infections and inevitable death in the vast majority of untreated patients [4].

Unfortunately, there is no effective AIDS vaccine currently available, and antiretroviral therapy is limited in its ability to fully control viral replication in infected individuals. Recent progress suggests that understanding how HIV interacts with the host immune cells is vitally important for the development of new treatments and effective vaccination regimens [1,2]. Monocytes, monocyte-differentiated dendritic cells (DCs) and macrophages are critical immune cells responsible for a wide range of both innate and adaptive immune functions [5]. These cell types also play multifaceted roles in HIV pathogenesis (Table 1). In this review, the potential roles of monocytes and DCs as HIV reservoirs and in latency will be discussed in detail.

**Table 1:** Myeloid lineage cell types and their potential roles and proposed mechanisms in HIV-1 latency

**Cell types**	**Primary Locations**	**Cellular markers**	**Potential role in HIV latency and proposed mechanisms**	**References**
Monocytes	Peripheral blood	CD14^++^ or CD16^+^CD14^+^	YES, but possibly mainly in CD16^+ ^cells • Restricted HIV-1 replication at different steps of viral life-cycle • Low molecular weight APOBEC3G (CD16^+ ^only) • Low level or undetectable Cyclin T1 • Impaired phosphorylation of CDK9	[10-12,87-92,94]

Macrophages	Mucosal surface/tissues	CD14^-^ EMR1^+^ CD68^+^	NO • High level Cyclin T1 • Phosphorylation of CDK9 and active P-TEFb	[14,18,94,97]

Myeloid DCs	Peripheral blood (immature) Lymph node (mature)	CD11c^+^ CD123^-^ BDCA1^+^	YES • Low level virus replication • Lymph node biopsies reveal presence • Unknown mechanism	[101,107,112]

Plasmacytoid DCs	Peripheral blood (immature) Lymph node (mature)	CD11c^-^ CD123^+^ BDCA2^+^ BDCA4^+^	Unlikely • Inhibiting HIV-1 replication through the secretion of IFNα and an unidentified small molecule • Unknown mechanism	[49,50,101]

Langerhans cells	Mucosal surface and epidermal tissue	CD1a^+^ Langerin^+^	Unlikely • Langerin inhibits virus transmission and enhances virus take-up and degradation • May act differently in co-infections	[40,41,113]

EMR1, epidermal growth factor module-containing mucin-like receptor 1 (a G-protein coupled receptor); BDCA, blood DC antigen.

## Monocytes interact with HIV-1

### Monocyte distribution and function

Monocytes are vitally important cells in the immune system, as they are the precursor cells to professional antigen-presenting cells (APCs), such as macrophages and DCs. These types of immune cells patrol the bloodstream and tissues, replenishing dying APCs or, in an infection, providing enough of these cells for the body to effectively combat an invading pathogen [5]. Undifferentiated monocytes live for only a few days in the bloodstream. Upon differentiation or activation, the life-span of monocytes is significantly prolonged for up to several months [6].

There are two major subtypes of monocytes, those that are highly CD14-positive (CD14^++^CD16^-^) and those that are CD16-positive (CD14^+^CD16^+^). CD16^+ ^cells make up only a small percentage (around 5%) of the total monocyte population, but they are characterised as more pro-inflammatory and having a greater role in infections than the CD14^++^CD16^- ^cells [7].

### HIV infection of monocyte

Although monocytes express the required HIV-1 receptors and co-receptors for productive infection [8,9], they are not productively infected by HIV-1 *in vitro*. This is possibly due to an overall inefficiency in each of the steps required for virus infection, ranging from viral entry to proviral DNA integration [10-12], but not due to a viral nucleocapsid uncoating defect [13]. Recent studies have suggested a role for naturally occurring anti-HIV micro-RNA (miRNA) in suppressing HIV-1 replication in peripheral blood mononuclear cells or purified monocytes [14-17]. This mechanism could allow for further studies utilising miRNAs as inhibitors of HIV-1 [15]. However, it has also been shown that HIV-1 is capable of suppressing some inhibitory miRNAs [16], which may reflect an evolutional interaction between HIV-1 and host factors. Further studies are required to understand this interaction and develop a therapeutic approach against HIV-1 infection using miRNAs.

Differentiation of monocytes into macrophages or DCs *in vitro *enables productive HIV-1 replication in the differentiated cells [14,18,19]. Based on current understanding, vaginal macrophages are more monocyte-like than intestinal macrophages and show increased HIV-1 susceptibility [20]. Hence, some monocyte characteristics might be required for efficient infection, and these traits may be lost in fully differentiated tissue macrophages.

### Monocyte-HIV interactions that impact immune function

Given the role of monocytes in the immune system and in HIV-1 replication, a number of HIV-1 proteins have been shown to affect the biology of monocytes.

HIV-1 Tat-mediated transactivation of the viral promoter is essential for HIV-1 transcription [21]. Exogenous recombinant HIV-1 Tat protein has been shown to increase monocyte survival through increased expression of the anti-apoptotic protein Bcl-2 [22]. Using an *in vitro *model of monocyte death mediated by TRAIL (tumour necrosis factor-alpha-related apoptosis inducing ligand), it has been shown that HIV-1 Tat encourages the survival of monocytes in situations where they would normally be cleared [22]. Exogenous HIV-1 Tat has been shown to cause production of the cytokine interleukin (IL)-10 from monocytes *in vitro *[23,24]. Significantly increased IL-10 levels were also observed in HIV/AIDS patients compared with healthy controls [25]. Furthermore, up-regulation of IL-10 production in HIV/AIDS patients has been correlated with increased levels of monocyte-secreted myeloid differentiation-2 and soluble CD14 [25]; both proteins are key molecules in the immune recognition of gram-negative bacterial lipopolysaccharide (LPS). Given that high levels of secreted CD14 have been associated with impaired responses to LPS [26], it has been proposed that the release of general immunosuppressant IL-10 by monocytes [27] facilitates the progression to AIDS [25].

HIV-1 Nef is a multifunctional accessory protein that plays an important role in viral pathogenesis [28]. Retroviral-mediated HIV-1 Nef expression in primary monocytes and a promonocytic cell line inhibits LPS-induced IL-12p40 transcription by inhibiting the JNK mitogen-activated protein kinases [29]. As an inducible subunit of biologically active IL-12, IL-12p40 plays a critical role in the development of cellular immunity, and its production is significantly decreased during HIV-1 infection [29]. This study implicates the importance of HIV-1 Nef in the loss of immune function and progression to AIDS.

HIV-1 matrix protein (p17) regulates a number of cellular responses and interacts with the p17 receptor (p17R) expressed on the surface of target cells [30]. Upon binding to the cell surface receptor p17R, exogenous HIV-1 matrix protein causes secretion of the chemokine monocyte chemotactic protein-1 (MCP-1, also known as CCL2) from monocytes [30]. MCP-1 potentially increases monocyte recruitment to the sites of HIV-1 infection, increasing the available monocyte pool for infection by HIV-1; this recruitment may be of critical importance given the relatively low rate of infection of this cell type [10-12].

HIV-1 and HIV-1-derived factors have been shown also to induce up-regulation of programmed death ligand-1 on monocytes *in vitro *[31,32]. This ligand, in complex with its receptor, programmed death-1, causes apoptosis of all T cell types [33] and a loss of anti-viral function in a manner similar to known immunosuppressive cytokines [34]. Together, these studies suggest that HIV-1 can impair virus-specific immunity by modulating immuno-regulatory molecules of monocytes and T cells.

Of the studies discussed above, those involving Tat, matrix protein and HIV-1-derived factors, were performed using recombinant or purified proteins, whereas the Nef study and the reports on the programmed death ligand-1 were performed using infectious viruses and *nef*-deleted HIV-1 mutants. Although these results shed light on the influence of individual viral proteins on monocytes *in vitro*, synergistic or antagonistic effects of HIV-1 proteins on cellular responses cannot be ruled out, nor can the roles played by other host factors *in vivo *be excluded.

**Figure 1:**
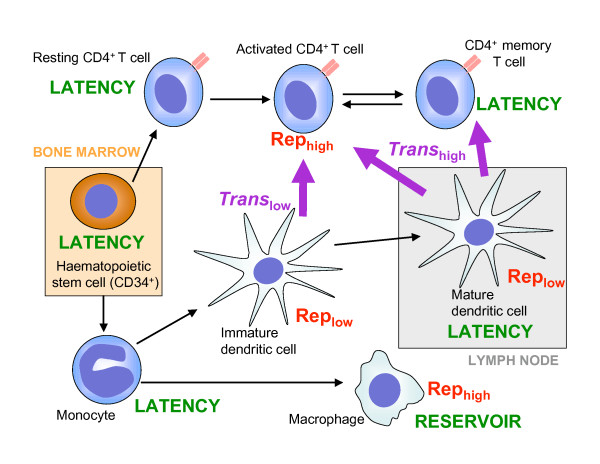
**Locations of HIV-1 replication and latency and routes of transmission between haematopoietic cell populations**. All cell types shown are susceptible to HIV-1 entry and integration of the proviral DNA. Some anatomical locations are shown; those outside of marked areas are in the bloodstream, lymphatic system and/or tissues. Black arrows represent differentiation and/or maturation and may represent more than one step and could involve multiple intermediate cell types. Purple arrows represent routes of *trans *infection, and relative rates are shown as high or low. "Rep" indicates productive HIV-1 replication with relative rates shown as high or low. HIV-1 *cis *infection routes are not shown, as any susceptible cell may be infected by productive replication from another cell. Those cells in which HIV-1 latency is thought to occur should be considered as putative viral reservoirs and therapeutic targets.

Overall, HIV-1 appears to promote the survival of monocytes as a key step for viral persistence. The interactions between the virus and monocytes may contribute key functions in establishing chronic HIV-1 infection and facilitating the progression to AIDS. These outcomes are likely influenced by the altered immunological function of monocytes and their interactions with other types of HIV-1 target cells (Figure 1).

## DCs interact with HIV

### Immune function of DCs

DCs are professional APCs that are differentiated from monocytes in specific cytokine environments. DCs bridge the innate and adaptive immune responses, as they endocytose and break down invading pathogens in the endolysosome or proteasome and present antigen fragments to T cells, usually in the context of major histocompatability complexes [5]. There are three major DC subtypes: myeloid DCs, plasmacytoid DCs (pDC), and Langerhans cells. These DC subtypes are characterised based on their locations, surface markers and cytokine secretion profiles [5].

DC life-span and survival are highly dependent on their anatomical locations and the DC subtypes [35]. In general, DC half-lives measure up to a few weeks, and they can be replaced through proliferating hematopoietic progenitors, monocytes, or tissue resident cells [35]. It has been shown that productive HIV-1 replication occurs in human monocyte-derived DCs for up to 45 days [36]. DCs may survive longer within the lymph nodes due to cytokine stimulation in the microenvironment, which may help spread HIV-1 infection and maintain viral reservoirs.

### HIV infection of DCs

HIV-1 is capable of directly infecting different DC subtypes (known as *cis *infection), but at a lower efficiency than HIV-1's ability to infect activated CD4^+ ^T cells; therefore, only a small percentage of circulating DCs are positive for HIV in infected individuals [19]. Productive HIV-1 replication is dependent on fusion-mediated viral entry in monocyte-derived DCs [37], and mature HIV-1 particles are localised to a specialised tetraspanin-enriched sub-compartment within the DC cytoplasm [38].

Langerhans cells are present in the epidermis or mucosal epithelia as immune sentinels [39]. It is interesting that Langerhans cells have been shown to be resistant to HIV-1 infection [40]. This resistance appears to be due to the expression of Langerin, which causes internalisation and break-down of HIV-1 particles and blocks viral transmission [40]. However, in the context of co-infection with other sexually transmitted organisms, such as the bacterium *Neisseria gonorrhoeae *and/or the fungus *Candida albacans *[41] or when stressed by skin abrasion [42], Langerhans cells can become more susceptible to HIV-1 infection and are able to transmit HIV-1 to CD4^+ ^T cells effectively [42].

Drug abuse can significantly facilitate HIV infection, transmission and AIDS progression through drug-mediated immunomodulation. Recent studies have suggested that the recreational drug, methamphetamine, increases susceptibility of monocyte-derived DCs to HIV-1 infection *in vitro *[43] and blocks the antigen presentation function of DCs [44]. Although its relevance to the *in vivo *situation is unclear, this finding is potentially a further risk factor (aside from the use of contaminated needles, etc.) associated with drug use and may explain the high levels of HIV-prevalence among drug abusers.

HIV-1 infection of DCs likely contributes to viral pathogenesis. Notably, HIV-2 is much less efficient than HIV-1 at infecting both myeloid DCs and pDCs, whilst retaining its infectivity of CD4^+ ^T cells [45]. This observation offers an explanation for the decreased pathogenicity of HIV-2, since HIV-2 will need to infect CD4^+ ^T cells directly and, perhaps more importantly, resting or memory CD4^+ ^T cells to ensure long-term survival of the virus.

### DC-HIV interactions that impact the immune function

Given the important roles DCs play in the immune response, it is reasonable that HIV-1 proteins or the virus itself have been shown to affect the function of DCs *in vitro*. Both HIV-1 matrix and Nef proteins have been shown to cause only partial maturation of pDCs *in vitro *[46,47]. In the presence of these viral proteins, DCs acquire a migratory phenotype, facilitating travel to the lymph nodes. However, these DCs do not express increased levels of activation markers, such as the T cell co-stimulatory molecules CD80 and CD86, or MHC class II, that would lead to a protective immune response [46,47]. It is possible, therefore, that the DCs are trapped in the lymph nodes and unable to initiate a protective immune response against the virus. The study of Nef protein's effects on DCs [47] was performed using a mouse DC model *in vitro *and an immortalised cell line; hence the full relevance of this finding to the *in vivo *situation is unclear.

Conversely, recombinant Nef protein appears to cause DC activation and differentiation by up-regulating the expression of CD80, CD86, MHC class II and other markers, as well as various cytokines and chemokines associated with T cell activation [48]. These effects have led to the proposition that Nef protein is capable of causing bystander activation of T cells via DCs [48], although this activity has not been demonstrated experimentally. Of note, the above study was performed using recombinant Nef alone.

DCs could contribute largely to an anti-HIV innate immunity. It has been demonstrated that pDCs are capable of inhibiting HIV-1 replication in T cells when cultured together *in vitro *[49,50], implicating the importance of pDCs for viral clearance. HIV-1 infected individuals are known to have lower levels of circulating pDCs compared with those of uninfected individuals [51]. It has been confirmed that HIV-1 is capable of directly killing pDCs [49], illustrating that the virus can remove a potential block to its replication and dissemination in pDCs.

HIV-1 can block CD4^+ ^T cell proliferation or induce the differentiation of naive CD4^+ ^T cells into T regulatory cells through pDCs [52,53]. These mechanisms involve HIV-1-induced expression of indoleamine 2,3-deoxygenase in pDCs. Indoleamine 2,3-deoxygenase is a CD4^+ ^T cell suppressor and regulatory T cell activator [52,53]. HIV-1 envelope protein gp120 has also been shown to inhibit activation of T cells by monocyte-derived DCs [54], suggesting that gp120 may also have a role in the suppression of T cell function and progression to AIDS.

In addition, HIV-1 has been shown to suppress the immune function of pDCs in general by suppressing activation of the anti-viral toll-like receptor 7 (TLR7) and TLR8 [55], and by blocking the release of the anti-viral interferon alpha [56]. A recent study indicated that divergent TLR7 and TLR9 signalling and type I interferon production in pDCs contribute to the pathogenicity of simian immunodeficiency virus (SIV) infection in different species of macaques [57]. These results suggest that chronic stimulation of pDCs by SIV or HIV in non-natural hosts may induce immune activation and dysfunction in AIDS progression [57]. Overall, HIV-1 inhibits the function of pDCs to allow maintenance of the virus within the host.

### DC-mediated HIV-1 trans infection

The most interesting aspect of HIV-1 infection in DCs is the ability of the cells to act as mediators of *trans *infection of activated CD4^+ ^T cells, which is the most productive cell type for viral replication. DC-mediated HIV-1*trans *infection of CD4^+ ^T cells is functionally distinct from *cis *infection [58,59] and involves the trafficking of whole virus particles from the DCs to the T cells via a 'virological synapse' [59,60]. Previous reviews have summarised the understanding of HIV-DC interactions [19,61]; so here we focus on discussing the latest progress in this field.

DC-mediated HIV-1 *trans *infection of CD4^+ ^T cells is dependent on, or enhanced by, a number of other cellular and viral factors. CD4 co-expression with DC-SIGN (DC-specific intercellular adhesion molecule 3-grabbing nonintegrin), a C-type lectin expressed on DCs, inhibits DC-mediated *trans *infection by causing retention of viral particles within the cytoplasm [62]. HIV-1 Nef appears to enhance DC-mediated HIV-1 *trans*-infection. Nef-enhanced HIV-1 transmission efficiency correlates with significant CD4 down-regulation in HIV-1-infected DCs [62]. Furthermore, the maturation state of the DCs appears to be important for *trans *infection, with mature DCs showing greater HIV trafficking ability than immature DCs [59,63-65]. These results have highlighted the proposed model that immature DCs might endocytose the virus in the periphery and then transfer it to CD4^+ ^T cells upon DC maturation in the lymph node [19].

Recent studies have revealed that the precise trafficking of the endocytosed HIV virion, with regard to the sub-cellular vesicle trafficking networks [64] and cytoskeletal rearrangements associated with synapse formation [63], is critical for *trans *infection in mature DCs. The host cell-derived glycosphingolipid composition of the viral particle also appears to be important for both the capture of virus in mature and immature DCs and the *trans *infection process [66]. Our recent results suggest that intracellular adhesion molecule-1 (ICAM-1), but not ICAM-2 or ICAM-3, is important for DC-mediated HIV-1 transmission to CD4^+ ^T cells [67]. The interaction between ICAM-1 on DCs and leukocyte function-associated molecule 1 (LFA-1) on T cells plays an important role in DC-mediated HIV-1 transmission [68]. This mechanism might be specific for DC-mediated transmission of HIV-1 to CD4^+ ^T cell, as *in vitro *experiments blocking LFA-1 on HIV-infected CD4^+ ^T cells have shown no effect on virus transmission to non-infected T cells [69]. In addition, purified host surfactant protein A in the mucosa has been shown to enhance DC-mediated HIV-1 transfer by binding to the viral envelope glycoprotein, gp120 [70]. This study also showed that surfactant protein A inhibited the direct infection of CD4^+ ^T cells [70], suggesting a selection pressure for DC-mediated *trans *infection at mucosal surfaces.

The precise mechanism of virus transfer from DCs to CD4^+ ^T cells has yet to be determined [19]. Recent studies have demonstrated a role for small lipid vesicles known as exosomes in immature and mature DC-mediated HIV-1 transmission to CD4^+ ^T cells [66,71,72]. Immature DCs are capable of constitutively releasing infectious virus in association with exosomes in the absence of CD4^+ ^T cells [71]. HIV-1 and purified exosomes can be endocytosed by mature DCs into the same intracellular compartment and transferred to co-cultured CD4^+ ^T cells [72], suggesting that HIV-1 may exploit an intrinsic exosome trafficking pathway in mature DCs to facilitate viral dissemination. Although interesting for infectious dynamics, these observations on exosome-mediated viral transmission do not sufficiently explain the mechanisms of HIV-1 *trans *infection. How these models relate to the *in vivo *situation of DC-mediated HIV-1 transmission is unclear, given that DCs can traffic to the lymph node and effectively transfer virus to CD4^+ ^T cells [19]. If DCs release HIV-1 in association with exosomes in the tissue as DCs migrate to lymph nodes [71], or if DCs require T cell activation for the release of exosome-associated HIV-1 [72], the viral transmission process might be very inefficient *in vivo*.

Recent studies have also offered the intriguing possibility that HIV-1 can be transferred from cell to cell via cell protrusions, with the virus either transmitting via cellular membrane nanotubes [73] or 'surfing' along the extracellular surface of the cytoplasmic membrane [74]. HIV-1 intracellular trafficking is dependent on the viral envelope protein on the membrane of an infected cell to form a stable complex with the protrusion from an uninfected cell [73]. This mechanism of viral transmission may be an adaptation of a normal cellular cross-talk process that is used in normal cellular communication, for example, by DCs and T cells during immunological synapse formation. Limitations to the above studies are that they were performed in either CD4^+ ^T cells alone [73], immortal CD4^+ ^T cells [74], or mainly using a mouse retroviral model [74]. Indeed, the potential mechanisms of cell-cell-mediated HIV transmission have yet to be investigated in the DC-T cell *trans *infection model.

Inhibition of cell-cell mediated HIV-1 transmission can be developed into future therapeutic approaches. Because of the importance of DC-mediated *trans *infection of CD4^+ ^T cells, a number of recent studies have identified factors that block this process, such as the C-type lectin, Mermaid, and natural anti-DC-SIGN antibodies in breast milk [75-78]. However, the therapeutic efficacy of these factors has yet to be established.

HIV-2 is incapable of being transferred from DCs [45]; and, coupled with its overall lack of *cis *infection of DCs, these data may explain why HIV-2 is less pathogenic than HIV-1.

## Potential role of monocytes and DCs in HIV-1 latency and reservoirs

In general, latency refers to the absence of gene expression of a pathogen in the infected hosts or cells, serving to ensure the long-term survival of the pathogen. Latency is an important step for a number of viral pathogens including HIV and other retroviruses [79-82]. Latency allows for the release of new viruses over an extended period of time and avoids short-term immune responses. The site of latency can form a viral reservoir, from which a virus can initiate new infections of naïve cells.

The critical aspect for supporting a viral reservoir is a cell type that will stay alive for a long time in order to preserve the virus. It has been shown that even with anti-retroviral therapy, low levels of HIV-1 viremia are maintained within the plasma of patients for at least 7 years [83]. Given that HIV-1 causes CD4^+ ^T-cell depletion and compromised immunological functions associated with AIDS [84], most CD4^+ ^T-cells are not sufficient for long-term maintenance of the virus. However, long-lived memory CD4^+ ^T cells can play an important role in HIV-1 latency [85,86]. This reservoir can persist for a long time during antiretroviral treatment; indeed, one study has suggested a viral half-life of 44 months [86], and another recent study showed survival of virus in the reservoir for 8.3 years without significant viral mutation [85]. These results suggest that the viral reservoir is protected from antiretroviral treatment and that it is capable of initiating new infections when the treatment is stopped.

Both monocytes and certain subsets of DCs have also been proposed as sites of HIV-1 latency (Figure 1 and Table 1).*In vivo *or *ex vivo *studies of HIV latency are generally performed using clinical samples from infected individuals undergoing antiretroviral therapy. The antiretroviral therapy may clear any easily accessible replicating virus and allow study of only the long-term HIV-l reservoirs.

### Role of monocytes

Monocytes are implicated as a viral reservoir based on the detection of, or the recovery of, infectious virus from monocytes isolated from HIV-positive individuals on antiretroviral therapy [87-91]. It appears that CD16-positive monocytes (5% of monocyte population [7]) are both more susceptible to infection and preferentially harbour the virus long-term [92,93], perhaps explaining why only small numbers of monocytes are infected by HIV-1 *in vitro*. CD14^++ ^monocytes express high levels of the low molecular weight form of APOBEC3G (apolipoprotein B mRNA-editing enzyme, catalytic polypeptide-like 3G), which is associated with anti-HIV activity, whereas CD16^+ ^monocytes express the high molecular weight form of APOBEC3G that has no anti-HIV activity [92].

The mechanism of HIV-1 latency in monocytes is not fully understood. Recent data suggest that the inhibition of viral replication is host mediated, at least in part, through a lack of the expression of key co-factors for the HIV-1 Tat protein. It appears that the transcription of the integrated viral genome, as transactivated by the viral Tat protein, is inhibited [94]. Tat binds to the 5' long terminal repeat sequence of the integrated genome in complex with two host proteins, cyclin T1 (CycT1) and cyclin-dependent kinase 9 (CDK9), collectively known as the positive transcription elongation factor b (P-TEFb) [21,95,96]. Monocytes, when compared with activated CD4^+ ^T cells and macrophages [96], are known to have much lower levels of CycT1 expression [94,97], therefore, they lack functional P-TEFb. However, this is not the only factor responsible for the resistance of monocytes to HIV-1 replication, as transient expression of CycT1 is not sufficient to restore HIV-1 Tat-mediated transactivation in monocytes [94]. Cell-cell fusion of monocytes and a HIV-1-permissive cell line restores Tat-mediated transactivation [94]. Phosphorylation of CDK9 is known to be vital for the formation of a P-TEFb complex and for Tat-mediated transcription of the HIV-1 promoter [98]. Despite having the same levels of CDK9, monocytes have low levels of the active, phosphorylated CDK9 form as compared with macrophages, and this phenotype has been directly correlated with the poor ability of monocytes to support HIV-1 replication [94]. In addition, the basal transcription from the HIV-1 LTR in undifferentiated primary monocytes was reported to be undetectable using a transient transfection assay [94].

Studying HIV-1 latency in monocytes is challenging due to generally low viral integration and infection of monocytes [94]. However, even when a HIV-1 proviral DNA construct is transfected directly into monocytes, there is no infectious virus production [94]. When monocytes differentiate into macrophages, they become increasingly susceptible to HIV-1 infection and permissive to viral gene expression and production of infectious viruses [94]. Furthermore, the differentiation of monocytes into macrophages stimulates HIV-1 production in the infected monocytes [94], suggesting a role played by monocytes in both viral latency and reactivation.

### Contribution of DCs

Because of the ability of DCs to transfer virus to CD4^+ ^T cells, it is conceivable that DCs may act as reservoirs for HIV-1 and 'dose' T cells with the virus over extended periods. DCs are capable of transmitting HIV-1 to T cells over a period of several days, and the viral transmission is dependent on viral replication [99-101]. It is possible, therefore, that long-term transfer of HIV-1 to T cells is actually through *cis *infection, while *trans *infection is only present in the very early stages [58]. This HIV-1 transmission process may be '*trans*-like', for example HIV-1 may assemble in endosomes or other intracellular membrane domains in a similar manner as described in macrophages [102,103], then the virus may be transmitted across a virological synapse. However, the precise mechanism of virus assembly within macrophages remains a source of debate [104,105].

The ability of DCs to act as reservoirs of HIV-1 appears to be highly dependent on the DC sub-type. Follicular DCs (FDCs) have been shown to retain infectious viral particles on their surface, and the retained virus is capable of being transferred to CD4^+ ^T cells [106-110]. FDCs in HIV-1 positive individuals harbour genetically diverse viral strains that are not observed elsewhere in the body [111], indicating that these cells may act as focal points for the rapid emergence of mutations observed in HIV-1 infected individuals.

It also appears that peripheral blood myeloid DCs do not harbour the virus *in vivo *during antiretroviral therapy [112], suggesting that it is the DCs in the lymph nodes that act as the long-term reservoir. This thinking is further supported by other studies that found HIV-1 in association with myeloid DCs that were isolated from lymph node biopsies or necropsies of individuals on antiretroviral therapy [107]. Conversely, a recent study has suggested that Langerhans cells isolated from the oral cavity of HIV-1 positive individuals do not act as reservoirs for HIV-1, despite HIV-1 detection within whole tissue samples from the area [113]. This result is perhaps not surprising given the effect of Langerin on inhibiting HIV-1 transmission [40]. Moreover, pDCs have also not been implicated as reservoirs of HIV-1, which may be due to inhibiting HIV-1 replication through the secretion of IFNα and an unidentified small molecule by pDCs [49,50].

### Role of monocytic precursor cells

HIV-1 is capable of altering the biology of haematopoietic stem cells *in vivo*, primarily affecting T cell development [114-116]. Undifferentiated monocytic precursor cells, such as CD34^+ ^stem cells or partially differentiated haematopoietic precursor cells, may act as reservoirs [117,118]. These cells in the bone marrow will be relatively shielded from antiviral treatments and may act as the ultimate long-term reservoir of HIV-1 (Figure 1). This mechanism allows for transmission of the virus because the progenitor cells containing integrated HIV-1 genomes will proliferate, differentiate and pass on the virus to progeny monocytes. Indeed, the ability to harbour genes and transfer them to progeny cells makes stem cells attractive targets for gene therapy against HIV-1 infection [119,120].

### Other proposed mechanisms

There have been a number of studies that have proposed other mechanisms for latency in CD4^+ ^memory T cells. It is possible that these mechanisms also have roles in latency in monocytes and/or DCs, but this remains to be investigated.

It has been proposed that the host cell itself can play a role through inhibition of HIV-1 gene transcription. In a CD4^+ ^T cell line and a yeast model of HIV-1 transcription, host chromatin structures slowly accumulate (in one study over 30 days [121]) on the long terminal repeat of the integrated viral genome and inhibit viral gene transcription [121,122]. Moreover, recent studies have suggested a much broader role for host transcription factors in HIV-1 latency in CD4^+ ^T cells [123-125].

In light of the evidence that suggests miRNAs play a role in the resistance of monocytes to HIV-1 infection [14,15], it is of interest that a number of host miRNAs have been implicated in causing latency in resting primary CD4^+ ^T cells [126]. Inhibitors of these miRNAs are now being touted as a new generation of treatment to be used in concert with current antiretrovirals [reviewed in [127]].

In resting CD4^+ ^T cells from HIV-1-infected individuals, HIV-1 multiply spliced RNA transcripts are retained in the nucleus and cannot be translated into functional proteins [128]. The lack of a host transcription factor, polypyrimidine tract binding protein, appears to account for the underlying mechanism in resting CD4^+ ^T cells. Transient expression of this host protein induces productive HIV-1 replication in resting CD4^+ ^T cells that are isolated from HIV-1-positive individuals [128].

However, HIV-1 latency is not always restricted to resting CD4^+ ^T cells or explained by limiting cellular factors. In some instances, HIV-1 latency is due to replicative selection for specific viral characteristics. It has been shown that a doxycycline-dependent HIV-1 variant is capable of establishing latency within a dividing CD4^+ ^T cell type (SupT1 cell line) normally permissive for viral replication [129]. This study showed that only a small proportion (0.1%–10%) of an inducible provirus was rescued from the cells after addition of the inducing doxycycline drug, indicating that HIV-1 is capable of establishing latency in the majority of actively dividing cells. Thus, in some settings, HIV-1 proviral latency is not limited to resting T cells, but can be due to intrinsic viral traits [129].

## Conclusion and future directions

Latency in HIV infection is a key area of study for understanding the pathogenesis and ultimate development of therapies or vaccinations against HIV/AIDS. Figure 1 shows an overview of the known or proposed interactions between HIV-1 and various cells of the haematopoietic system. Moreover, myeloid lineage cell types and their potential roles and proposed mechanisms in HIV-1 latency are summarized in Table 1.

Efforts to tackle HIV latency may ultimately fall into two key areas, blocking the development of the latency and reactivating viral reservoirs in chronically infected individuals to clear the virus. Both aspects will require extensive understanding of the mechanisms of HIV latency [1,2]. Given that monocytes and DCs have been implicated as HIV-1 reservoirs using *in vitro *and *ex vivo *models of viral infection (Table 1), further understanding of the mechanisms of latency within these cells is an important area of research. Although much is known about the ways in which HIV-1 interacts with both monocytes and the various types of DCs, some key questions remain to be answered to fully understand the pathogenesis and latency of HIV-1. For instance, the relative contributions of the proposed cell types in the process of HIV latency and molecular mechanisms in both viral and host aspects remain to be elucidated.

The latent phase is of particular interest for the development of novel anti-HIV interventions. The HIV and host-factor interactions described here represent potential targets for both drug and vaccination efforts. Given that HIV-1 has a very intimate relationship with host cells, blocking known host factors responsible for certain viral effects could have catastrophic consequences for the host. For example, blocking DC factors responsible for virological synapse formation may also switch off the formation of the immunological synapses that arise in response to HIV or other pathogen infections. The ultimate hope would be to find either a viral factor or non-essential host factors that can be removed without damage to the host. As a successful example, the CCR5 co-receptor is now a target of both HIV-1 gene therapy and antiretroviral therapy [130,131]. Based on studies into the role of DCs in HIV-1 pathogenesis, there are also a number of post-exposure vaccine clinical trials, wherein DCs are exposed *ex vivo *with HIV-1 or HIV-1 antigens and then re-introduced into the HIV-positive individual in an effort to elicit a protective immune response [reviewed in [132]].

Development of *in vitro *models of HIV-1 latency can be extremely complex. While there are examples of complex tissue culture models of *in vivo *systems for a range of human pathogens, including HIV-1, these models involve predominantly epithelial cells and various leukocytes [133,134]. Cell culture-based models containing only subsets of leukocytes have limitations, because it is impossible to compartmentalise the cells in exactly the same fashion as observed *in vivo *(as in lymph nodes, for example). There are also many important technical issues with isolation, maintenance and establishment of *in vitro *studies of HIV-1 latency [reviewed in [135]].

*In vivo *or *ex vivo *model systems remain the best options for studying long-term HIV-1 latency. SIV strains that are closely related to HIV and display the same initial infection and latency characteristics can be used as attractive models to study viral latency. Mice are generally not susceptible to HIV-1, or at least not in a physiologically relevant manner. Recently, 'humanised' mice have become available in HIV-1 research [reviewed in [136]]. The humanised mouse model potentially offers a viable alternative to non-human primates for studying HIV-1 molecular pathogenesis and for designing novel therapies that block HIV-1 infection [137].

## Abbreviations

HIV-1: human immunodeficiency virus type 1; HIV-2: human immunodeficiency virus type 2; SIV: simian immunodeficiency virus; DCs: dendritic cells; pDC: plasmacytoid DCs; APCs: antigen-presenting cells; CD: cluster of differentiation; IL: interleukin; LPS: lipopolysaccharide; TLR: toll-like receptor.

## Competing interests

The authors declare that they have no competing interests.

## Authors' contributions

Both authors contributed to the writing and editing of the manuscript.
